# Transcranial direct current stimulation of the primary motor cortex improves word-retrieval in older adults

**DOI:** 10.3389/fnagi.2014.00253

**Published:** 2014-09-23

**Authors:** Marcus Meinzer, Robert Lindenberg, Mira M. Sieg, Laura Nachtigall, Lena Ulm, Agnes Flöel

**Affiliations:** ^1^Center for Clinical Research, The University of QueenslandHerston, QLD, Australia; ^2^Department of Neurology, NeuroCure Clinical Research Center, Charité University MedicineBerlin, Germany; ^3^Department of Neurology, Center for Stroke Research Berlin, Charité University MedicineBerlin, Germany

**Keywords:** transcranial direct current stimulation, functional magnetic resonance imaging, language, motor, aging

## Abstract

Language facilitation by transcranial direct current stimulation (tDCS) in healthy individuals has generated hope that tDCS may also allow improving language impairment after stroke (aphasia). However, current stimulation protocols have yielded variable results and may require identification of residual language cortex using functional magnetic resonance imaging (fMRI), which complicates incorporation into clinical practice. Based on previous behavioral studies that demonstrated improved language processing by motor system pre-activation, the present study assessed whether tDCS administered to the primary motor cortex (M1) can enhance language functions. This proof-of-concept study employed a sham-tDCS controlled, cross-over, within-subject design and assessed the impact of unilateral excitatory (anodal) and bihemispheric (dual) tDCS in 18 healthy older adults during semantic word-retrieval and motor speech tasks. Simultaneous fMRI scrutinized the neural mechanisms underlying tDCS effects. Both active tDCS conditions significantly improved word-retrieval compared to sham-tDCS. The direct comparison of activity elicited by word-retrieval vs. motor-speech trials revealed bilateral frontal activity increases during both anodal- and dual-tDCS compared to sham-tDCS. This effect was driven by more pronounced deactivation of frontal regions during the motor-speech task, while activity during word-retrieval trials was unaffected by the stimulation. No effects were found in M1 and secondary motor regions. Our results show that tDCS administered to M1 can improve word-retrieval in healthy individuals, thereby providing a rationale to explore whether M1-tDCS may offer a novel approach to improve language functions in aphasia. Functional magnetic resonance imaging revealed neural facilitation specifically during motor speech trials, which may have reduced switching costs between the overlapping neural systems for lexical retrieval and speech processing, thereby resulting in improved performance.

## Introduction

The language and motor action systems feature tight functional connections and share neural resources (Willems and Hagoort, 2007). They are organized in partially overlapping neural networks where higher order cortices can be involved in a flexible, context-dependent manner in different functions (Bressler and Menon, 2010; Behrens and Sporns, 2012). In the context of language production, the cortico-bulbar system controls muscles involved in speech and breathing. Furthermore, cortico-subcortical loops comprising primary (M1) and non-primary motor areas as well as the ventrolateral thalamus and striatum are not only involved in the initiation and sequencing of speech, but also in cognitive control processes (Crosson, 2013; Dick et al., 2013). Besides reciprocal cortico-subcortical interplay, cortical motor areas are strongly connected with inferior frontal regions that are crucial for word-retrieval processes and also the selection of motor actions (Thompson-Schill et al., 1997; Pobric and Hamilton, 2006; Eickhoff et al., 2009).

While there is an on-going debate about the origin and extent of language-motor system interactions and also the (linguistic) level at which the motor system impacts on language processing, the mutual interplay between the two systems is generally not questioned (Rizzolatti and Craighero, 2004; Willems and Hagoort, 2007; Pulvermuller and Fadiga, 2010). The behavioral relevance of these interactions is illustrated by studies showing that lexical retrieval and semantic processing can be facilitated by execution or observation of manual gestures (Hadar et al., 1998; Holle and Gunter, 2007; Dick et al., 2009) and prohibiting manual gestures can slow down speech production (Rauscher et al., 1996; Pine et al., 2007). Pre-activation of the motor system by different behavioral interventions also improved lexical retrieval in patients with post-stroke aphasia (Hanlon et al., 1990; Harnish et al., 2011; Meinzer et al., 2011a; Benjamin et al., 2014). So far, however, the neural mechanisms by which the motor system facilitates language production remain largely elusive.

Motor cortex excitability can be modulated by non-invasive transcranial direct current stimulation (tDCS; Stagg and Nitsche, 2011), providing a promising tool to alter interactions between motor and language systems. During tDCS, weak electrical currents are administered to the scalp that exert local effects on the underlying cortex, but also on functionally connected remote regions (Miniussi et al., 2013; Flöel, 2014). Excitatory tDCS (anodal-tDCS) facilitates motor learning when applied to M1 contralateral to the hand involved in a task (Reis et al., 2009) and these effects may be even more pronounced with simultaneous inhibitory (cathodal) tDCS to the ipsilateral M1 (dual-tDCS, (Vines et al., 2008)). In the language domain it has been shown that anodal-tDCS administered to perisylvian regions of the left language dominant hemisphere improved language processing (Flöel, 2012; Monti et al., 2013) and learning (de Vries et al., 2010; Meinzer et al., 2014a). Moreover, inhibitory tDCS of M1 impaired learning of a novel lexicon (Liuzzi et al., 2010). However, so far, it is unknown if facilitation of M1 by anodal-tDCS can improve linguistic processing.

Therefore, the present randomized, sham-controlled study employed a within-subjects design to study effects of M1 stimulation on semantic word-generation. Given that M1-tDCS has been suggested as an adjunct treatment approach for post-stroke motor (Lindenberg et al., 2010, 2012) and language rehabilitation (Pulvermüller and Berthier, 2008; Meinzer et al., 2011a), and that stroke typically affects older people, the present proof-of-concept study assessed the impact of M1-tDCS in healthy older adults. We combined tDCS with simultaneous functional magnetic resonance imaging (fMRI) to assess its effects on task performance and brain activity (Meinzer et al., 2014b). Using the same task, we have previously demonstrated that anodal-tDCS administered to the left inferior frontal gyrus facilitated word-retrieval and selectively reduced task-related activity in bilateral prefrontal cortices during word-retrieval, but not during a simple motor speech baseline task (Meinzer et al., 2013). However, given that the exact (linguistic) level at which the motor system impacts on language processing is still under scrutiny (Willems and Hagoort, 2007) and that M1 stimulation may affect functional brain activity in premotor and prefrontal regions (Antal et al., 2011; Lindenberg et al., 2013), we explored whether potential tDCS effects on performance would be mediated by modulations of motor and/or language regions during either or both of these tasks.

## Methods

### Study overview

Data was acquired in the context of a study that assessed the impact of M1 stimulation on motor and linguistic performance and brain functions during resting-state and task-related fMRI. In a sham-tDCS controlled, within-subject, triple cross-over design, participants were scanned during three MRI sessions with simultaneous intrascanner tDCS. Active stimulation was administered during two of these sessions, either unilaterally to the left M1 (anodal-tDCS) or bilaterally (dual-tDCS). During dual-tDCS, anodal-tDCS was administered to the left M1, while cathodal-tDCS was administered simultaneously to the right M1 (Lindenberg et al., 2013).

**Figure 1:**
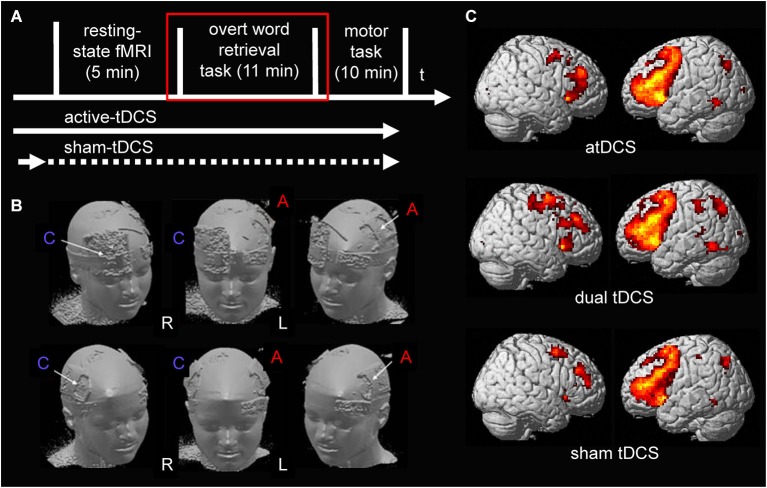
**(A)** Illustrates the design of the present study. tDCS was ramped up prior to the start of the resting-state scan during all stimulation conditions. During sham-tDCS the current was ramped down after 30 s (before scanning commenced). During both active stimulation conditions (anodal- and dual-tDCS), it continued until after the end of the word-retrieval task (red square; only data acquired during the word-retrieval task is reported). **(B)** Surface rendering illustrates the location of the electrodes during the two active stimulation conditions on the scalp: upper row shows anodal-tDCS montage; lower row shows dual-tDCS montage. Please note, the larger size of the reference electrode (cathode) during anodal-tDCS renders the stimulation over the supraorbital cortex functionally ineffective. R = right hemisphere, L = left hemisphere. C = Cathode, A = Anode. **(C)** Surface rendering of activity patterns elicited by semantic word-retrieval vs. motor speech trials during the three stimulation conditions. Overall, as in previous studies that used the same task, this contrast elicited mainly activity in bilateral lateral frontal and medial frontal and premotor regions. Right column = Left hemisphere, Left column = Right hemisphere. All contrasts were thresholded at *p* < 0.05, family-wise-error corrected at voxel and cluster levels.

During each session, participants first completed a resting-state scan followed by two subsequent task-related fMRI scans (an overt semantic word-retrieval task and a motor choice reaction task, Figure 1A illustrates the design of the study). The three scanning sessions were separated by approximately 1 week to prevent potential carry over effects of the active stimulation conditions and the order of stimulation conditions was counterbalanced across subjects. Please note, only data acquired during the semantic word generation task is reported here. The impact of the three stimulation conditions on resting-state functional connectivity and activity elicited by the motor choice reaction task have previously been reported (Lindenberg et al., 2013). The experimental set-up was identical to previous cross-over studies of our group that assessed the impact of anodal-tDCS administered to left perisylvian language areas on semantic word-retrieval (Meinzer et al., 2012a, 2013). The study was approved by the ethics committee of the Charité University Hospital, and conducted in accordance with the Helsinki declaration. Written informed consent was obtained from all subjects prior to study inclusion.

### Subjects

Eighteen healthy older adults participated in this study (9 women, 9 men; mean age 68.38 ± 5.15 years). Details of the sample have been reported previously (for details see Lindenberg et al., 2013 and Table 1). In short, all participants presented with age-appropriate cognitive status (as assessed by the test battery established by The Consortium to Establish a Registry for Alzheimer’s Disease, CERAD-Plus^1^), and structural imaging parameters. They were right-handed according to the Edinburgh Handedness Inventory (mean ± SD 91 ± 15, Oldfield, 1971), participated for the first time in a tDCS study, had no history of current or previous neurological or psychiatric disorders and did not report use of psychoactive medication or recreational drugs.

**Table 1:** **Demographic characteristics of the participants and details of the neuropsychological assessment (mean and standard deviation, raw data)**.

	Mean ± SD	Range
**Age** (years)	68.38 ± 5.15	61–77
**Education** (years)	15.88 ± 4.74	11–19
**Sex** (females/males)	9/9
**CERAD subtests**
**Mini mental state examination**	29.44 ± 0.62	28–30
**Verbal fluency**
(# examplars produced in one minute)
Semantic fluency	25.61 ± 9.41	14–31
Phonemic fluency	16.39 ± 4.37	12–25
**Boston naming test**	14.67 ± 0.59	13–15
**Verbal learning and memory test** (# correct)
Learning success (sum score runs 1–3)	20.44 ± 3.55	16–27
Delayed recall	7.89 ± 1.84	6–10
**Visual spatial items** (sum score)
Copy	10.83 ± 0.70	8–11
Delayed recall	10.7 ± 2.62	7–14
**Trail making test A**
(time to completion, s)	39.94 ± 7.07	31–49
**Trail making test B**	74.00 ± 20.86	42–103

### Transcanial direct current stimulation

A constant direct current (1 mA) was administered by an MRI-compatible stimulator (DC-Stimulator Plus®, NeuroConn, Ilmenau, Germany) using an established set-up (for details see Meinzer et al., 2014b). The anode was placed inside a 5 × 7 cm^2^ saline-soaked sponge pocket and attached over the left M1 in all stimulation conditions (C3 of the 10–20 EEG system) as described in our previous manuscript (Lindenberg et al., 2013). For dual stimulation, the cathode (5 × 7 cm^2^) was placed over the right M1 (position C4). During anodal-tDCS the reference electrode (10 × 10 cm^2^) was positioned over the right supraorbital region. Those electrode montages represent the most commonly used set-ups for unihemispheric anodal-tDCS and dual-tDCS of the motor cortex. During sham-tDCS the reference electrode was pseudo-randomly assigned to either the right supraorbital region or right M1 in half of the participants to counterbalance those two montages across the group. Figure 1B illustrates the respective montages.

In all stimulation conditions, the current was initially increased to 1 mA in a ramp-like fashion over 10 s shortly prior to the start of the RS-sequence and remained constant for 30 min during anodal-tDCS and dual-tDCS, thereby covering the entire duration of the language task (which took approximately 11 min, see below). During sham-tDCS, the current was turned off after 30 s prior to the start of the RS-sequence. In all stimulation conditions, the current was ramped down over 10 s at the end of the stimulation.

### fMRI task and stimulus characteristics

Magnetic resonance imaging data were acquired using a 3-Tesla Siemens Trio MR scanner at the Berlin Center for Advanced Neuroimaging (Charité University Hospital, Berlin, Germany). The overt semantic word-retrieval task was identical as in previous studies of our group (Meinzer et al., 2012a, 2013) and employed a T2*-weighted echo-planar imaging (EPI) sequence (TR/TA = 6000/2000 ms, TE = 30, flip angle: 90°, 32 transverse slices, gap: 0.75 mm, interleaved acquisition, FOV: 192 × 192, acquisition matrix: 64 × 64, 104 volumes) and a temporal sparse sampling design. This allows assessing overt verbal responses during a scanner off phase to avoid articulation related artifacts. Six semantic categories (6 blocks of 10 consecutive trials of the same category, trial duration 3.8 s) were presented using a projector and a system of mirrors. Participants were instructed to overtly produce one different exemplar during each trial or say “next” in case they could not come up with a response. In between trials, a black screen was displayed (2.2 s) and the hemodynamic response was acquired (sparse sampling). Task blocks alternated with a simple motor speech baseline condition (saying the word “rest”; five consecutive trials) in response to a written cue. Eighteen pre-selected semantic categories were used that were divided into three matched sets based on published norms (Set_1_: trees, insects, sports equipment, body parts, beverages, occupations; Set_2_: flowers, fish, kitchen appliances, clothing, food, hobbies, Set_3_: spices, birds, toys, colors, auto parts, musical instruments; Sets_1/2/3_: total # exemplars produced in norm group: 1586/1587/1650, average category size: 11.6/11.8/12.2; fluency: 0.64/0.60/0.59, all *p* > 0.0.92; Mannhaupt, 1983). In addition, 20 different subjects participated in a pilot study that assessed performance using these 18 categories during a standard semantic verbal fluency task (duration: 1 min; categories were presented in randomized order). The number of exemplars produced was comparable between the three sets (mean ± SD # correct exemplars produced Set_1_: 19.38 ± 6.5; Set_2_: 19.28 ± 6.4; Set_3_:19.31 ± 6.2; *p* = 0.98). The three sets were counterbalanced across the group. Prior to scanning, participants were trained using a different set of categories. During scanning, overt responses were recorded using an MRI-compatible microphone and transcribed for subsequent analysis. The scoring of responses was performed by two raters blinded to the stimulation conditions using recorded and transcribed responses. The number of correct responses was determined according to guidelines established during previous studies of our group (Meinzer et al., 2012a, 2013). Repeated measures analysis of variance (RM-ANOVA) assessed performance differences between stimulation conditions.

### fMRI data analysis

Statistical Parametric Mapping (SPM5, Welcome Department of Imaging Neuroscience, London, UK) was used for data analysis. Pre-processing of the data was identical as in our previous studies (Meinzer et al., 2012a, 2013) and comprised re-alignment of functional images, co-registration with the individual participants’ anatomical images, unified segmentation and registration to MNI space, and spatial smoothing (8 × 8 × 8 mm^3^ Gaussian kernel). Covariates-of-interest (correct word-retrieval and motor speech baseline condition trials) and movement parameters were included in the design matrix. Afterwards, a high-pass filter (128 s) was applied, data were modelled with a finite impulse response and the contrasts-of-interest were estimated. Those included the comparison of:

1. Correct word-retrieval vs. motor speech trials.
2. Word-retrieval or motor speech trials vs. the implicit baseline as implemented in SPM (Meinzer et al., 2013). The latter were modelled separately to explore potential differential effects of the stimulation on both tasks.

As in our previous study (Lindenberg et al., 2013), we employed an *a priori* region-of-interest (ROI) approach using the Anatomy Toolbox (Eickhoff et al., 2005). It was previously demonstrated that the semantic word-generation task mainly elicits activity in bilateral frontal cortices in older adults (Meinzer et al., 2009, 2012b,c, 2013), therefore, left and right Brodmann Areas (BA) 44 and 45 were chosen as *a priori* ROIs to assess the impact of tDCS on language processing. These areas overlap with the anterior (BA45) and posterior (BA44) portions of the inferior frontal gyrus that is tightly connected with the motor system (Eickhoff et al., 2009; Pulvermuller and Fadiga, 2010). These areas also overlapped with regions showing peak activity during the three fMRI sessions in the bilateral frontal cortex. Figure 1C illustrates the activity pattern elicited by the task during the three stimulation conditions, activity patterns surviving a family-wise corrected voxel and cluster threshold of *p* < 0.05 are shown. Repeated measures analysis of variance (ANOVA) assessed differences between mean beta activity elicited by the tasks (complex contrast word-retrieval vs. baseline trials; separate comparisons of both tasks vs. the implicit baseline) during the three stimulation conditions in the four frontal ROIs (Greenhouse-Geisser corrected results are reported). Results of *post hoc* paired *t*-tests were corrected for multiple comparisons using the false discovery rate (FDR; Benjamini and Hochberg, 1995). Pearson correlation coefficients tested whether potential stimulation induced performance improvements would be associated with activity changes in ROIs.

Please note, in response to a reviewer’s request during the revision of our previous study (Lindenberg et al., 2013), the impact of the stimulation conditions on activity elicited by the *word-retrieval task* in M1 (BA4) and premotor (BA6) regions for the complex contrast (word-generation > motor speech baseline condition trials) has already been reported. No activity differences were found between the stimulation conditions. However, as for bilateral frontal ROIs, we also explored whether tDCS would impact selectively on task or baseline trials during the word-generation task (Meinzer et al., 2013). This was not the case and we therefore do not report details of this analysis.

## Results

All participants tolerated the stimulation well and no adverse effects were noted. A post-study questionnaire indicated that participants could not differentiate between the stimulation conditions, therefore, effective blinding was achieved by this set-up (for details see Lindenberg et al., 2013).

### Impact of tDCS on performance

**Figure 2:**
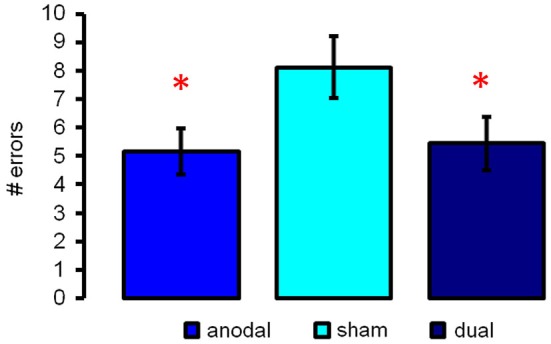
**Illustrates semantic word-retrieval performance during the three stimulation conditions (anodal-, sham- and dual-tDCS)**. Both active stimulation conditions improved performance as compared to sham; no significant differences were found between anodal- and dual-tDCS. Data show mean ± SEM # of errors (max. 60), * *p* < 0.05.

Repeated measures ANOVA revealed significant differences between the three stimulation conditions (*F*_(2,16)_ = 7.74, *p* = 0.0004). *Post hoc* paired *t*-tests showed that both active stimulation conditions resulted in superior performance during the semantic word-retrieval task, indicated by a smaller number of errors (anodal-tDCS vs. sham-tDCS: *t*_(17)_ = 3.37, *p* = 0.0036, Cohen’s *d* = 0.795; dual tDCS vs. sham: *t*_(17)_ = 3.62, *p* = 0.0021, Cohen’s *d* = 0.853, Figure 2, both effects remained significant after FDR-correction *p* < 0.022). Performance was comparable in the two active stimulation conditions (17) = 0.33, *p* = 0.74).

### ROI analysis (word-retrieval vs. motor speech trials, Figure 3A)

**Figure 3:**
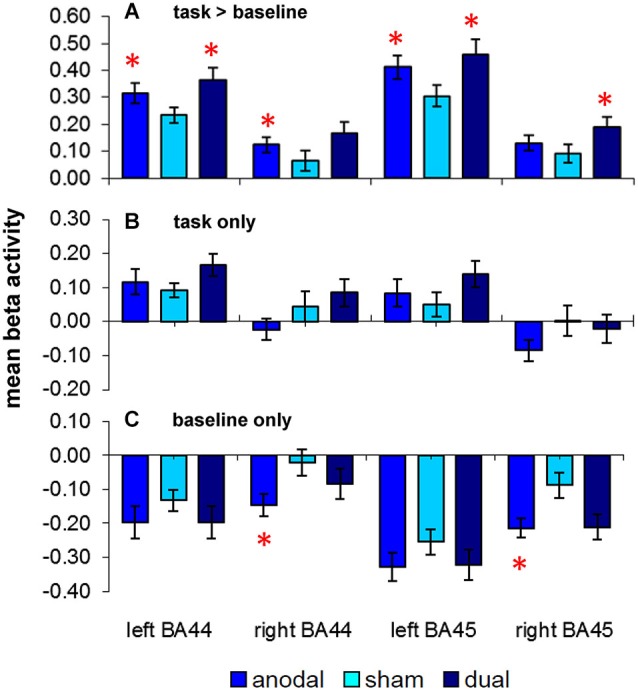
**Results of the *a priori* region-of-interest (ROI) analysis in bilateral frontal regions (BAs 44/45): (A)** upper panel depicts activity elicited by the semantic word-retrieval task as compared to motor speech trials (complex contrast) during the three stimulation conditions (anodal-, sham-, dual-tDCS). Lower panels show separate comparisons of activity elicited by **(B)** word-retrieval and **(C)** motor speech trials with the implicit baseline. Data show mean ± SEM, *p < 0.05 FDR-corrected corrected for multiple comparisons.

Repeated measures ANOVA revealed a significant effect of STIMULATION (*F*_(1.2,19.5)_ = 9.5, *p* = 0.004). *Post hoc* paired *t*-tests showed that mean beta activity in both left-sided ROIs was significantly higher during both active stimulation conditions as compared to sham-tDCS (left BA 44/45 anodal-tDCS > sham-tDCS: *t*_(17)_ = 3.29/4.40, *p* = 0.017/0.004; dual-tDCS > sham-tDCS: *t*_(17)_ = 2.88/3.92, *p* = 0.031/0.006). Anodal-tDCS compared to sham-tDCS resulted in increased activity in right BA44 (*t*_(17)_ = 2.52, *p* = 0.044), the comparison of dual-tDCS vs. sham-tDCS in this ROI did not survive FDR-correction (*t*_(17)_ = 2.29, *p* = 0.06). However, activity in right BA45 was selectively enhanced during dual-tDCS compared to sham-tDCS (*t*_(17)_ = 2.7, *p* = 0.033). The main effect of ROI was also significant (*F*_(1.6,26.5)_ = 37.5, *p* < 0.001), however, this is explained by larger beta values in both left sided ROIs during all stimulation conditions as expected for a left lateralized language task. The interaction STIMULATION × ROI was not significant (*p* = 0.439). Therefore, active stimulation resulted in enhanced activity in bilateral frontal ROIs. No linear correlations between changed task-related activity and improved performance were found.

### ROI analysis (word-retrieval/motor speech trials vs. implicit baseline, Figure 3B)

Two additional RM-ANOVAs assessed whether word-retrieval or motor speech trials were differentially affected by the respective stimulation conditions. For *word-retrieval trials*, these analyses revealed no significant effects of STIMULATION (*F*_(1.6,27.7)_ = 1.67, *p* = 0.21). The interaction of STIMULATION × ROI approached significance (*F*_(2.50,42.6)_ = 2.95, *p* = 0.052), however, none of the *post hoc* comparisons survived an FDR-corrected threshold. Therefore, significant stimulation effects in the comparison above (word-retrieval vs. motor speech trials) were not driven by a direct effect on bilateral frontal activity during word-retrieval trials.

For *motor speech trials* (Figure 3C), a different pattern emerged. Overall, negative beta values in all ROIs across stimulation conditions indicate that those areas are deactivated during the simple motor speech baseline. Moreover, the degree of deactivation was more pronounced during both active stimulation conditions. Repeated measures ANOVA revealed that the main effect of ROI was significant (*F*_(2.2,36.9)_ = 27.99, *p* < 0.001) and the effect of STIMULATION approached significance (*F*_(1.8,31.5)_ = 3.26, *p* = 0.055). Two *post hoc* comparisons survived an FDR-corrected threshold and revealed a significantly greater decrease of activity during anodal-tDCS compared to sham-tDCS in right BAs 44 and 45 (*t*_(17)_ = 3.03/2.91, both *p* > 0.02). As for the complex contrast (word-retrieval vs. motor speech trials), no linear correlations between changes in activity and performance were found. Despite numerically larger deactivations in all ROIs during dual-tDCS, no significant differences were found compared to sham-tDCS.

In sum, enhanced activity in bilateral frontal ROIs during active-tDCS for the complex contrast (word-retrieval vs. motor speech trials) were mainly explained by more pronounced deactivations in these areas during motor speech trials, while the stimulation did not affect word-retrieval trials.

## Discussion

The results of this proof-of-concept study demonstrate that both anodal and dual-tDCS administered to M1 can improve word-retrieval in healthy older adults. Simultaneous fMRI allowed unprecedented insights into the neural mechanisms mediating tDCS-induced behavioral facilitation. Specifically, improved performance during both active stimulation conditions was accompanied by up-regulation of bilateral prefrontal activity as compared to sham-tDCS during the complex contrast of word-retrieval vs. motor speech baseline trials, while no stimulation-effects were found in primary or secondary motor cortices. This effect was mainly driven by more pronounced deactivation of frontal regions during the motor speech baseline task. Given that the human brain operates on limited neural resources (Brem et al., 2014), this may have facilitated switching between the two overlapping neural systems involved in both tasks and in turn explain improved behavioral performance during semantic word-retrieval trials. With regard to clinical applications, the positive behavioral results of this study provide a rationale to explore whether M1 stimulation can enhance treatment outcome in patients with aphasia, thereby providing an exciting novel “backdoor” approach to facilitate language recovery after stroke (Pulvermüller and Berthier, 2008; Meinzer et al., 2011a).

### Impact of tDCS on word-retrieval

Previous studies that have administered anodal-tDCS to anterior or posterior left-sided perisylvian language regions in healthy individuals reported beneficial stimulation effects during language production (Iyer et al., 2005; Cattaneo et al., 2011; Meinzer et al., 2012a, 2013) and language learning paradigms (de Vries et al., 2010; Meinzer et al., 2014a). Those findings have generated hope that tDCS may be suited to enhance language recovery in patients with post-stroke language impairment. Indeed, a number of studies have provided preliminary evidence that anodal-tDCS administered to perilesional areas in the left hemisphere (Baker et al., 2010; Fridriksson et al., 2011) and also contralesional brain regions (Flöel et al., 2011) may enhance the effectiveness of simultaneous speech therapy. Similarly, dual-tDCS to prefrontal regions enhanced treatment effects in aphasia as well (Marangolo et al., 2013, 2014). However, perilesional tDCS requires a pre-treatment fMRI scan to identify residual language cortex in individual patients, which complicates its incorporation in routine clinical practice. Furthermore, up to 40% of treated patients may not benefit from perilesional stimulation (Baker et al., 2010). Alternative stimulation sites that do not require pre-treatment fMRI scans (e.g., contralesional areas) also yielded variable effects and so far, there is no consensus which areas should be stimulated in individual patients (Meinzer et al., 2011b; Holland and Crinion, 2012). This highlights the need to explore alternative stimulation sites that are easy to implement and also effective in clinical practice.

Stimulation of motor areas that are anatomically and functionally linked to the language system (Dick et al., 2013) may represent an exciting novel approach to facilitate language processing in aphasia (Pulvermüller and Berthier, 2008; Meinzer et al., 2011a). A number of behavioral studies in healthy individuals and patients with aphasia have shown that pre-activation of the motor system can improve language production (Hanlon et al., 1990; Hadar et al., 1998; Holle and Gunter, 2007; Dick et al., 2009; Meinzer et al., 2011a; Benjamin et al., 2014). Moreover, inhibition of M1 by cathodal-tDCS resulted in reduced language learning (Liuzzi et al., 2010). Closer inspection of behavioral improvements reported in aphasia patients after perilesional stimulation in the study by Baker et al. (2010) also revealed that the majority of responsive patients received anodal-tDCS to premotor regions that are tightly connected to M1. In addition, a recent cross-over sham-tDCS controlled single case report reported significantly improved naming ability during anodal-tDCS administered to the left primary motor cortex in a patient with aphasia (Datta et al., 2011). Our results are also in line with recent studies demonstrating that language-motor system interactions may not be limited to action-specific language materials (e.g., verbs describing motor related actions like walking or throwing) but generalize to non-action related verbs and objects (Liuzzi et al., 2008; Meinzer et al., 2011a; Postle et al., 2013). Therefore, the positive results of our study provide a rationale to assess whether M1 stimulation can enhance treatment outcome in patients with post-stroke aphasia. Given that no major differences were found between anodal- and dual-tDCS with regard to performance and brain activity patterns, it is plausible that tDCS effects in the present study are mainly mediated by the left-hemispheric anodal component that was administered in both active stimulation conditions.

### Impact on task-related activity

A large number of behavioral studies have demonstrated beneficial effects of anodal and dual-tDCS on cognition, language and motor functions (Flöel, 2012, 2014; Monti et al., 2013; Kuo et al., 2014). However, only a handful of studies have combined tDCS with simultaneous fMRI to elucidate the neural underpinnings of these effects (Saiote et al., 2013; Meinzer et al., 2014b). For example, in the motor domain, Antal et al. (2011) demonstrated that anodal-tDCS administered to M1 during a finger tapping task resulted in reduced task-related activity in the pre-supplementary motor area while no effects were found at the stimulation site. Similarly, Lindenberg et al. (2013) did not find activity changes in bilateral M1 after anodal-tDCS administered to the motor cortex, while resting-state fMRI revealed connectivity changes in distant areas, including bilateral prefrontal regions. Therefore, cortico-spinal excitability changes during M1-tDCS not always translate into changes in regional brain activity changes at the stimulation site as measured by fMRI but may affect functionally connected remote brain regions. Similarly, M1 stimulation did not impact on task-related activity in primary and secondary motor cortices during the word-retrieval task in the present study, however, stimulation effects were found in functionally connected bilateral IFG (Eickhoff et al., 2009).

Previous studies that employed word-retrieval tasks during anodal-tDCS of left perisylvian regions improved picture naming and semantic word-generation performance in healthy older adults which was associated with reduced task-related activity in left-sided (Holland et al., 2011; Meinzer et al., 2012a) or bilateral IFG (Meinzer et al., 2013). In those studies, reduced activity was interpreted as more efficient processing due to tDCS-induced neural facilitation. Moreover, using the same task as in the present study, this effect could be explained by selectively reduced activity during word-retrieval trials, but not during simple motor speech trials (Meinzer et al., 2013). In the present study, we show that improved word-retrieval after M1-tDCS was mediated by a different mechanism. Specifically, active-tDCS did not impact on task-related activity during word-retrieval trials, but resulted in more pronounced deactivation of bilateral prefrontal activity during motor speech trials, which explains the net activity increase in those areas, emphasizing specific tDCS-effects on different tasks (Iuculano and Cohen Kadosh, 2013). Compared to sham-tDCS, the degree of prefrontal deactivation was numerically similar during both active stimulation conditions; however, it only reached significance in right-frontal regions during anodal-tDCS. Tentatively, this finding could be explained by higher current flow to right frontal regions during unilateral M1-tDCS with a supraorbital reference electrode as suggested by previous modelling studies (Wagner et al., 2007; Bikson et al., 2012; Kuo et al., 2013).

Funnelling of information that arises from different neural networks during dual task performance in the prefrontal cortex has been suggested to create a bottleneck for information processing and consequently lower behavioral output. Training can improve dual-task performance by inducing more efficient neural processing, typically expressed as reduced activity during fMRI in prefrontal cortex (Dux et al., 2009). Similarly, behavioral improvement in the present study may have been mediated by further disengagement (deactivation) of prefrontal regions during motor speech trials, possibly freeing processing resources or reducing switching costs between the two tasks that are engaging partially overlapping networks (Brem et al., 2014). However, this hypothesis needs to be scrutinized in future studies that allow examining interactions between the neural networks for speech production and lexical retrieval and their modulation by tDCS using different imaging paradigms and functional connectivity analysis. Such an analysis was not feasible in this present study due to the blocked-sparse sampling design. Moreover, brain regions in the vicinity of the targeted M1 may also undergo excitability changes due to the large size of the electrode or spill-over effects (Bestmann et al., 2004) and it is conceivable that stimulation effects may have extended into the neighboring parietal areas. Future studies are thus required to determine whether prefrontal activity changes during M1 stimulation are mediated by direct effects of M1 on interconnected prefrontal regions or by modulation of a larger fronto-parietal network.

## Conclusions

In sum, the present study demonstrates that M1 stimulation can improve word-retrieval in healthy older individuals and provides first evidence for the underlying neural mechanisms mediating behavioral facilitation. Our results also provide a rationale to explore the effectiveness of M1 stimulation as an alternative and clinically feasible adjunct treatment approach in post-stroke aphasia. In addition, our findings confirm that language-motor interactions may extend beyond the well-known impact on action-specific language material (Liuzzi et al., 2008; Meinzer et al., 2011a; Postle et al., 2013), which emphasizes its broad potential to enhance language recovery in clinical settings. Finally, the imaging results emphasize that different tDCS-montages may result in specific neural effects on different tasks that require further scrutiny.

## Conflict of interest statement

The authors declare that the research was conducted in the absence of any commercial or financial relationships that could be construed as a potential conflict of interest.

## Footnotes

^1^http://www.memoryclinic.ch

## References

[B1] Antal A. Polania R. Schmidt-Samoa C. Dechent P. Paulus W. (2011). Transcranial direct current stimulation over the primary motor cortex during fMRI. Neuroimage 55, 590–596 10.1016/j.neuroimage.2010.11.085 21211569

[B2] Baker J. M. Rorden C. Fridriksson J. (2010). Using transcranial direct-current stimulation to treat stroke patients with aphasia. Stroke 41, 1229–1236 10.1161/STROKEAHA.109.576785 20395612 PMC2876210

[B3] Behrens T. E. Sporns O. (2012). Human connectomics. Curr. Opin. Neurobiol. 22, 144–153 10.1016/j.conb.2011.08.005 21908183 PMC3294015

[B4] Benjamin M. L. Towler S. Garcia A. Park H. Sudhyadhom A. Harnish S. (2014). A behavioral manipulation engages right frontal cortex during aphasia therapy. Neurorehabil. Neural Repair 28, 545–553 10.1177/1545968313517754 24407914 PMC4090303

[B5] Benjamini Y. Hochberg Y. (1995). Controlling the false discovery rate: a practical and powerful approach to multiple testing. J. R. Statist. Soc. B 57, 289–300

[B6] Bestmann S. Baudewig J. Siebner H. R. Rothwell J. C. Frahm J. (2004). Functional MRI of the immediate impact of transcranial magnetic stimulation on cortical and subcortical motor circuits. Eur. J. Neurosci. 19, 1950–1962 10.1111/j.1460-9568.2004.03277.x 15078569

[B7] Bikson M. Rahman A. Datta A. Fregni F. Merabet L. (2012). High-resolution modeling assisted design of customized and individualized transcranial direct current stimulation protocols. Neuromodulation 15, 306–315 10.1111/j.1525-1403.2012.00481.x 22780230 PMC3418452

[B8] Brem A. K. Fried P. J. Horvath J. C. Robertson E. M. Pascual-Leone A. (2014). Is neuroenhancement by noninvasive brain stimulation a net zero-sum proposition? Neuroimage 85(Pt. 3), 1058–1068 10.1016/j.neuroimage.2013.07.038 23880500 PMC4392930

[B9] Bressler S. L. Menon V. (2010). Large-scale brain networks in cognition: emerging methods and principles. Trends Cogn. Sci. 14, 277–290 10.1016/j.tics.2010.04.004 20493761

[B10] Cattaneo Z. Pisoni A. Papagno C. (2011). Transcranial direct current stimulation over Broca’s region improves phonemic and semantic fluency in healthy individuals. Neuroscience 183, 64–70 10.1016/j.neuroscience.2011.03.058 21477637

[B11] Crosson B. (2013). Thalamic mechanisms in language: a reconsideration based on recent findings and concepts. Brain Lang. 126, 73–88 10.1016/j.bandl.2012.06.011 22831779 PMC3514571

[B12] Datta A. Baker J. M. Bikson M. Fridriksson J. (2011). Individualized model predicts brain current flow during transcranial direct-current stimulation treatment in responsive stroke patient. Brain Stimul. 4, 169–174 10.1016/j.brs.2010.11.001 21777878 PMC3142347

[B13] de Vries M. H. Barth A. C. Maiworm S. Knecht S. Zwitserlood P. Flöel A. (2010). Electrical stimulation of Broca’s area enhances implicit learning of an artificial grammar. J. Cogn. Neurosci. 22, 2427–2436 10.1162/jocn.2009.21385 19925194

[B14] Dick A. S. Bernal B. Tremblay P. (2013). The language connectome: new pathways, new concepts. Neuroscientist [Epub ahead of print]. 10.1177/1073858413513502 24342910

[B15] Dick A. S. Goldin-Meadow S. Hasson U. Skipper J. I. Small S. L. (2009). Co-speech gestures influence neural activity in brain regions associated with processing semantic information. Hum. Brain Mapp. 30, 3509–3526 10.1002/hbm.20774 19384890 PMC2896896

[B16] Dux P. E. Tombu M. N. Harrison S. Rogers B. P. Tong F. Marois R. (2009). Training improves multitasking performance by increasing the speed of information processing in human prefrontal cortex. Neuron 63, 127–138 10.1016/j.neuron.2009.06.005 19607798 PMC2713348

[B17] Eickhoff S. B. Heim S. Zilles K. Amunts K. (2009). A systems perspective on the effective connectivity of overt speech production. Philos. Trans. A Math. Phys. Eng. Sci. 367, 2399–2421 10.1098/rsta.2008.0287 19414462 PMC3268212

[B18] Eickhoff S. B. Stephan K. E. Mohlberg H. Grefkes C. Fink G. R. Amunts K. (2005). A new SPM toolbox for combining probabilistic cytoarchitectonic maps and functional imaging data. Neuroimage 25, 1325–1335 10.1016/j.neuroimage.2004.12.034 15850749

[B19] Flöel A. (2012). Non-invasive brain stimulation and language processing in the healthy brain. Aphasiology 26, 1082–1102 10.1080/02687038.2011.589892

[B20] Flöel A. (2014). tDCS-enhanced motor and cognitive function in neurological diseases. Neuroimage 85(Pt. 3), 934–947 10.1016/j.neuroimage.2013.05.098 23727025

[B66] Flöel A. Meinzer M. Kirstein R. Nijhof S. Deppe M. Knecht S. (2011). Short-term anomia training and electrical brain stimulation. Stroke 42, 2065–2067 10.1161/STROKEAHA.110.609032 21636820

[B21] Fridriksson J. Richardson J. D. Baker J. M. Rorden C. (2011). Transcranial direct current stimulation improves naming reaction time in fluent aphasia: a double-blind, sham-controlled study. Stroke 42, 819–821 10.1161/STROKEAHA.110.600288 21233468 PMC8210639

[B22] Hadar U. Wenkert-Olenik D. Krauss R. Soroker N. (1998). Gesture and the processing of speech: neuropsychological evidence. Brain Lang. 62, 107–126 10.1006/brln.1997.1890 9570882

[B23] Hanlon R. E. Brown J. W. Gerstman L. J. (1990). Enhancement of naming in nonfluent aphasia through gesture. Brain Lang. 38, 298–314 10.1016/0093-934x(90)90116-x 2322814

[B24] Harnish S. Meinzer M. Trinastic J. Fitzgerald D. Page S. (2011). Language changes coincide with motor and fMRI changes following upper extremity motor therapy for hemiparesis: a brief report. Brain Imaging Behav. [Epub ahead of print]. 10.1007/s11682-011-9139-y 21989635

[B25] Holland R. Crinion J. (2012). Can tDCS enhance treatment of aphasia after stroke? Aphasiology 26, 1169–1191 10.1080/02687038.2011.616925 23060684 PMC3464450

[B26] Holland R. Leff A. P. Josephs O. Galea J. M. Desikan M. Price C. J. (2011). Speech facilitation by left inferior frontal cortex stimulation. Curr. Biol. 21, 1403–1407 10.1016/j.cub.2011.07.021 21820308 PMC3315006

[B27] Holle H. Gunter T. C. (2007). The role of iconic gestures in speech disambiguation: ERP evidence. J. Cogn. Neurosci. 19, 1175–1192 10.1162/jocn.2007.19.7.1175 17583993

[B28] Iuculano T. Cohen Kadosh R. (2013). The mental cost of cognitive enhancement. J. Neurosci. 33, 4482–4486 10.1523/JNEUROSCI.4927-12.2013 23467363 PMC3672974

[B29] Iyer M. B. Mattu U. Grafman J. Lomarev M. Sato S. Wassermann E. M. (2005). Safety and cognitive effect of frontal DC brain polarization in healthy individuals. Neurology 64, 872–875 10.1212/01.wnl.0000152986.07469.e9 15753425

[B30] Kuo H. I. Bikson M. Datta A. Minhas P. Paulus W. Kuo M. F. (2013). Comparing cortical plasticity induced by conventional and high-definition 4 × 1 ring tDCS: a neurophysiological study. Brain Stimul. 6, 644–648 10.1016/j.brs.2012.09.010 23149292

[B31] Kuo M. F. Paulus W. Nitsche M. A. (2014). Therapeutic effects of non-invasive brain stimulation with direct currents (tDCS) in neuropsychiatric diseases. Neuroimage 85(Pt. 3), 948–960 10.1016/j.neuroimage.2013.05.117 23747962

[B32] Lindenberg R. Nachtigall L. Meinzer M. Sieg M. M. Flöel A. (2013). Differential effects of dual and unihemispheric motor cortex stimulation in older adults. J. Neurosci. 33, 9176–9183 10.1523/JNEUROSCI.0055-13.2013 23699528 PMC6705011

[B33] Lindenberg R. Renga V. Zhu L. L. Nair D. Schlaug G. (2010). Bihemispheric brain stimulation facilitates motor recovery in chronic stroke patients. Neurology 75, 2176–2184 10.1212/WNL.0b013e318202013a 21068427 PMC3013585

[B34] Lindenberg R. Zhu L. L. Schlaug G. (2012). Combined central and peripheral stimulation to facilitate motor recovery after stroke: the effect of number of sessions on outcome. Neurorehabil. Neural Repair 26, 479–483 10.1177/1545968311427568 22258156 PMC3666327

[B35] Liuzzi G. Ellger T. Flöel A. Breitenstein C. Jansen A. Knecht S. (2008). Walking the talk—speech activates the leg motor cortex. Neuropsychologia 46, 2824–2830 10.1016/j.neuropsychologia.2008.05.015 18606424

[B36] Liuzzi G. Freundlieb N. Ridder V. Hoppe J. Heise K. Zimerman M. (2010). The involvement of the left motor cortex in learning of a novel action word lexicon. Curr. Biol. 20, 1745–1751 10.1016/j.cub.2010.08.034 20888226

[B37] Mannhaupt H. R. (1983). Produktionsnormen für verbale Reaktionen zu 40 geläufigen Kategorien. Lang. Cogn. 4, 264–278

[B38] Marangolo P. Fiori V. Cipollari S. Campana S. Razzano C. Di Paola M. (2013). Bihemispheric stimulation over left and right inferior frontal region enhances recovery from apraxia of speech in chronic aphasia. Eur. J. Neurosci. 38, 3370–3377 10.1111/ejn.12332 23930827

[B39] Marangolo P. Fiori V. Gelfo F. Shofany J. Razzano C. Caltagirone C. (2014). Bihemispheric tDCS enhances language recovery but does not alter BDNF levels in chronic aphasic patients. Restor. Neurol. Neurosci. 32, 367–379 10.3233/RNN-130323 24398720

[B40] Meinzer M. Antonenko D. Lindenberg R. Hetzer S. Ulm L. Avirame K. (2012a). Electrical brain stimulation improves cognitive performance by modulating functional connectivity and task-specific activation. J. Neurosci. 32, 1859–1866 10.1523/JNEUROSCI.4812-11.2012 22302824 PMC6703352

[B41] Meinzer M. Breitenstein C. Westerhoff U. Sommer J. Rösser N. Rodriguez A. D. (2011a). Motor cortex preactivation by standing facilitates word retrieval in aphasia. Neurorehabil. Neural Repair 25, 178–187 10.1177/1545968310376577 20966157 PMC3594773

[B42] Meinzer M. Flaisch T. Seeds L. Harnish S. Antonenko D. Witte V. (2012b). Same modulation but different starting points: performance modulates age differences in inferior frontal cortex activity during word-retrieval. PLoS One 7:e33631 10.1371/journal.pone.0033631 22438970 PMC3305312

[B43] Meinzer M. Flaisch T. Wilser L. Eulitz C. Rockstroh B. Conway T. (2009). Neural signatures of semantic and phonemic fluency in young and old adults. J. Cogn. Neurosci. 21, 2007–2018 10.1162/jocn.2009.21219 19296728 PMC2730979

[B44] Meinzer M. Harnish S. Conway T. Crosson B. (2011b). Recent developments in functional and structural imaging of aphasia recovery after stroke. Aphasiology 25, 271–290 10.1080/02687038.2010.530672 21532927 PMC3083028

[B45] Meinzer M. Jähnigen S. Copland D. A. Darkow R. Grittner U. Avirame K. (2014a). Transcranial direct current stimulation over multiple days improves learning and maintenance of a novel vocabulary. Cortex 50, 137–147 10.1016/j.cortex.2013.07.013 23988131

[B46] Meinzer M. Lindenberg R. Antonenko D. Flaisch T. Flöel A. (2013). Anodal transcranial direct current stimulation temporarily reverses age-associated cognitive decline and functional brain activity changes. J. Neurosci. 33, 12470–12478 10.1523/JNEUROSCI.5743-12.2013 23884951 PMC6618670

[B47] Meinzer M. Lindenberg R. Darkow R. Ulm L. Copland D. Flöel A. (2014b). Transcranial direct current stimulation and simultaneous functional magnetic resonance imaging. J. Vis. Exp., e51730 10.3791/51730 24796646 PMC4181345

[B48] Meinzer M. Seeds L. Flaisch T. Harnish S. Cohen M. L. McGregor K. (2012c). Impact of changed positive and negative task-related brain activity on word-retrieval in aging. Neurobiol. Aging 33, 656–669 10.1016/j.neurobiolaging.2010.06.020 20696496 PMC2989336

[B49] Miniussi C. Harris J. A. Ruzzoli M. (2013). Modelling non-invasive brain stimulation in cognitive neuroscience. Neurosci. Biobehav. Rev. 37, 1702–1712 10.1016/j.neubiorev.2013.06.014 23827785

[B50] Monti A. Ferrucci R. Fumagalli M. Mameli F. Cogiamanian F. Ardolino G. (2013). Transcranial direct current stimulation (tDCS) and language. J. Neurol. Neurosurg. Psychiatry 84, 832–842 10.1136/jnnp-2012-302825 23138766 PMC3717599

[B51] Oldfield R. C. (1971). The assessment and analysis of handedness: the Edinburgh inventory. Neuropsychologia 9, 97–113 10.1016/0028-3932(71)90067-4 5146491

[B65] Pine K. J. Bird H. Kirk E. (2007). The effects of prohibiting gestures on children’s lexical retrieval ability. Dev. Sci. 10, 747–754 10.1111/j.1467-7687.2007.00610.x 17973791

[B52] Pobric G. Hamilton A. F. (2006). Action understanding requires the left inferior frontal cortex. Curr. Biol. 16, 524–529 10.1016/j.cub.2006.01.033 16527749

[B53] Postle N. Ashton R. McFarland K. de Zubicaray G. I. (2013). No specific role for the manual motor system in processing the meanings of words related to the hand. Front. Hum. Neurosci. 7:11 10.3389/fnhum.2013.00011 23378833 PMC3561662

[B54] Pulvermüller F. Berthier M. L. (2008). Aphasia therapy on a neuroscience basis. Aphasiology 22, 563–599 10.1080/02687030701612213 18923644 PMC2557073

[B55] Pulvermuller F. Fadiga L. (2010). Active perception: sensorimotor circuits as a cortical basis for language. Nat. Rev. Neurosci. 11, 351–360 10.1038/nrn2811 20383203

[B64] Rauscher F. H. Krauss R. M. Chen Y. (1996). Gesture, speech, and lexical access: the role of lexical movements in speech production. Psychol. Sci. 7, 226–231 10.1111/j.1467-9280.1996.tb00364.x

[B56] Reis J. Schambra H. M. Cohen L. G. Buch E. R. Fritsch B. Zarahn E. (2009). Noninvasive cortical stimulation enhances motor skill acquisition over multiple days through an effect on consolidation. Proc. Natl. Acad. Sci. U S A 106, 1590–1595 10.1073/pnas.0805413106 19164589 PMC2635787

[B57] Rizzolatti G. Craighero L. (2004). The mirror-neuron system. Ann. Rev. Neurosci. 27, 169–192 10.1146/annurev.neuro.27.070203.144230 15217330

[B58] Saiote C. Turi Z. Paulus W. Antal A. (2013). Combining functional magnetic resonance imaging with transcranial electrical stimulation. Front. Hum. Neurosci. 7:435 10.3389/fnhum.2013.00435 23935578 PMC3733022

[B59] Stagg C. J. Nitsche M. A. (2011). Physiological basis of transcranial direct current stimulation. Neuroscientist 17, 37–53 10.1177/1073858410386614 21343407

[B60] Thompson-Schill S. L. D’Esposito M. Aguirre G. K. Farah M. J. (1997). Role of left inferior prefrontal cortex in retrieval of semantic knowledge: a reevaluation. Proc. Natl. Acad. Sci. U S A 94, 14792–14797 10.1073/pnas.94.26.14792 9405692 PMC25116

[B61] Vines B. W. Cerruti C. Schlaug G. (2008). Dual-hemisphere tDCS facilitates greater improvements for healthy subjects’ non-dominant hand compared to uni-hemisphere stimulation. BMC Neurosci. 9:103 10.1186/1471-2202-9-103 18957075 PMC2584652

[B62] Wagner T. Fregni F. Fecteau S. Grodzinsky A. Zahn M. Pascual-Leone A. (2007). Transcranial direct current stimulation: a computer-based human model study. Neuroimage 35, 1113–1124 10.1016/j.neuroimage.2007.01.027 17337213

[B63] Willems R. M. Hagoort P. (2007). Neural evidence for the interplay between language, gesture and action: a review. Brain Lang. 101, 278–289 10.1016/j.bandl.2007.03.004 17416411

